# New graduate medication safety preparedness: an Australian cross-sectional and longitudinal qualitative research study

**DOI:** 10.3389/fmed.2026.1704787

**Published:** 2026-02-13

**Authors:** Ella Ottrey, Charlotte E. Rees, Kayley M. Lyons, Tina P. Brock, Lynn V. Monrouxe, Claire Harrison, Julia Morphet

**Affiliations:** 1 Monash Centre for Scholarship in Health Education (MCSHE), Faculty of Medicine, Nursing and Health Sciences, Monash University, Clayton, VIC, Australia; 2 Faculty of Medicine, Health and Life Science, Swansea University, Swansea, United Kingdom; 3 Faculty of Pharmacy and Pharmaceutical Sciences, Monash University, Parkville, VIC, Australia; 4 School of Population and Global Health, Faculty of Medicine, Dentistry and Health Sciences, University of Melbourne, Melbourne, VIC, Australia; 5 Collaborative Practice Centre, Faculty of Medicine, Dentistry and Health Sciences, University of Melbourne, Melbourne, VIC, Australia; 6 School of Health Sciences, Faculty of Medicine and Health, The University of Sydney, Camperdown, NSW, Australia; 7 School of Public Health and Preventive Medicine, Faculty of Medicine, Nursing and Health Sciences, Monash University, Melbourne, VIC, Australia; 8 Monash Nursing and Midwifery, Faculty of Medicine, Nursing and Health Sciences, Monash University, Clayton, VIC, Australia

**Keywords:** longitudinal qualitative research (LQR), medication administration, medication error, medication safety, new graduates, preparedness

## Abstract

**Introduction:**

Patient safety is paramount, yet medication management errors are common, including amongst new graduates. Ongoing need exists to examine new graduates’ medication safety preparedness, to better improve preparedness and help them manage medication errors. This cross-sectional and longitudinal qualitative research (LQR) explores new graduates’ medication safety preparedness in nursing, pharmacy and medicine.

**Methods:**

Underpinned by social constructionism, 26 final-year healthcare students at an Australian university participated in three study phases between July 2019 and April 2020: entrance interviews (around degree completion), longitudinal audio-diaries (through approximately the first 12 weeks of work), and exit interviews (after approximately 12 weeks of work). We analyzed interview and audio-diary transcripts, and audio-diary email correspondence using team-based framework analysis, cross-sectionally and longitudinally.

**Results:**

Participants’ medication safety stories demonstrated mostly unpreparedness, often about developing and implementing medication therapy plans. Medication error narratives revealed errors (of commission or omission) made by new graduates or others. They were rich in emotional talk (mostly negative such as anxiety, anger and sadness talk), illustrating psychosocial impacts on new graduates. However, positive emotional talk was also present in preparedness stories. While the proportion of preparedness stories increased across time at the cohort level, we found more nuanced/complex patterning in participants’ narratives at the individual level including evidence of stability, and positive or negative changes in medication safety preparedness.

**Discussion:**

We offer evidence-based recommendations for student/new graduate learning to help educators better prepare them for medication safety and enable them to cope with the emotional work of safe medication management. Further LQR with longer study durations is now needed on medication safety preparedness.

## Introduction

Patient safety–the prevention of adverse effects and avoidable errors–is paramount in healthcare (1). And yet, medication errors, both of commission (action) and omission (inaction) are common (2, 3). Numerous conceptualizations of preparedness for practice exist in the health professions education literature including preparedness as experience, preparedness as capabilities (e.g., knowledge, confidence, self-awareness, skills, competence, employability, self-initiative, responsibility/accountability, resilience) and/or preparedness as temporal (short or long-term) (4). While new graduates spend considerable time on medication administration processes (5–8), they often feel inadequately prepared for medication knowledge, prescribing, dispensing, administering and monitoring (5–7, 9–18). Consequently, medication errors involving new graduates are commonplace (8–10, 19–23). Despite burgeoning preparedness for practice literature in health professions education [e.g., (12, 14, 24–26)], insufficient literature still exists specifically examining in-depth new graduates’ preparedness for safe medication management. Therefore, this cross-sectional and longitudinal qualitative research (LQR) aims to bring depth, as well as longitudinal, and multiprofessional insights to explore new graduates’ medication safety preparedness. Without this work, we cannot fully understand how best to improve new graduates’ preparation for safe medication management, nor help them cope in the face of medication errors, for the betterment of new graduates and patients alike.

### Perceptions of medication management preparedness

New healthcare graduates report feeling underprepared for medication management (5, 7, 9–18). Indeed, diversity in confidence is evident across primary research and systematic reviews [e.g., (27)]. In nursing, while graduates within their first 2 years of practice report self-perceived strengths in various aspects of medication management (e.g., correct dosage, monitoring, identifying unsafe practices), understanding mechanism of action and drug clearance remains challenging (28). Furthermore, a recent longitudinal study of 42 nursing students participating in simulated scenarios across four semesters identified all five rights (drug, patient, dose, time, route) as incomplete in 80% of cases at graduation; suggesting that these graduating nurses were not competent in safe medication administration (7). In pharmacy, Malaysian pharmacy supervisors flagged new graduates’ suboptimal clinical reasoning skills (e.g., struggles applying medication knowledge to practice), and communication skills and confidence when interacting with colleagues and patients about medications (29). In medicine, although final-year students generally reported preparedness for responsible antibiotic prescribing (mean 71.2%), preparedness differed from 95.7% (recognizing clinical signs of infection) to 43.9% [communicating disagreement with senior doctors’ prescribing directives (30)]. Furthermore, junior doctors are thought to struggle with prescribing IV fluids, overlooking important tasks and/or enacting others incorrectly (11).

### Factors influencing preparedness for medication safety

The literature illustrates numerous factors influencing preparedness and unpreparedness for medication safety and errors, at the individual, relationship and organizational levels (5, 16, 18, 28, 31, 32). At the individual level, facilitators of medication safety preparedness for new graduates include nurses’ positive attitudes, subjective norms, perceived behavioral control and intentions to practice safe medication management [e.g., (33)], and new graduate pharmacists’ knowledge and skills facilitating safe medication practice [e.g., (34)]. Conversely, individual-level factors influencing unpreparedness highlight new graduates’ professional neglect in the face of medication alerts (19, 35), pharmacy graduates’ failure to engage actively during ward rounds and apply knowledge to practice (34), and suboptimal competence for medication administration; thought largely to be due to inadequate undergraduate education.

At the relationship level, factors influencing preparedness include junior doctors’ supportive interprofessional relationships, with praise and developmental feedback (32). While junior doctors report learning prescribing from observing and role-modeling senior colleagues’ prescribing practices (11, 21, 36), pharmacists also contribute to junior doctors’ prescribing practices through instruction, guidance and feedback (10, 11, 21, 31, 36). Conversely, relationship-level factors influencing unpreparedness include new graduates receiving insufficient supervision and feedback from seniors, with junior doctors reporting reluctance to seek advice (11, 21, 36). Junior doctors wanted more checking of their prescribing, and new graduate nurses wanted more information/advice about treatment side-effects (34).

Organizational-level factors influencing preparedness largely pertain to medication safety education, including workplace-based training as new graduates, and undergraduate education [e.g., (13, 18, 28)]. Regarding formal graduate workplace-based training (10, 11, 21, 31, 36), simulation activities (e.g., anaphylactic shock management) are shown to be effective in teaching new graduate nurses six competencies for managing anaphylaxis (37). In medicine, ward-based interprofessional teaching (e.g., pharmacist-led safe insulin prescribing sessions) improved junior doctors’ self-reported confidence and knowledge regarding safe insulin prescribing for patients living with diabetes (38). New medical graduates recommended pharmacist-led teaching, joint ward rounds and standardized communication to improve communication and increase them enacting pharmacists’ advice (31). Furthermore, junior doctors attending a vancomycin educational session or receiving pocket guidelines report higher self-confidence for drug monitoring and amending dosage (39). In undergraduate medical education, wide-ranging interventions have been identified for facilitating preparedness, including online, case-based, simulated, interprofessional, experiential, game-based and/or peer learning (15, 22). Furthermore, the online Prescribing Safety Assessment (PSA; and associated learning resources) for final-year medical students in the United Kingdom [with the international Prescribing Skills Assessment version: (3, 6, 20, 40, 41)] is thought to improve practical prescribing skills (42). Research suggests that a pharmacist-led prescribing intervention for final-year medical students (including pre-prescribing: students writing prescriptions and receiving pharmacist feedback) yields increased confidence, awareness of good prescribing practices and prescribing skills (13).

However, considerable literature illustrates education-related organizational-level factors for unpreparedness. For example, only a minority (36.4%) of 1023 medical students from 25 medical schools in the United Kingdom believed their undergraduate course prepared them adequately for prescribing, calling for earlier introduction of practical prescribing skills training (including simulation and pre-prescribing interventions) (42). Moreover, medical students typically report needing more antibiotic prescribing education through, for example, discussing clinical cases/vignettes, small group teaching, and infectious diseases clinical placements (30). New graduate doctors express wanting more focused undergraduate education on drug interactions, avoiding adverse drug reactions, prescribing for special patient groups, therapeutic drug monitoring, basic pharmacology and pharmacokinetics (23). Furthermore, while medical graduates may have prescribing knowledge, they report insufficient experiences with pharmacists during medical school, struggling with knowledge application (21). Finally, organizational-level factors influencing unpreparedness also include new graduates’ working conditions (34), including heavy workloads relating to medication management for nurses from the beginning of their roles (8), and considerable medication interruptions contributing to medication errors (6, 43, 44).

### Critique of literature, study aim and research questions

Previous studies have begun to articulate new graduates’ medication safety preparedness, alongside factors influencing preparedness and unpreparedness, yet this literature is broad, disparate and fragmented (hence the large number of citations in this paper). It is found across multiple professions, centered on various topics (e.g., preparedness, medication safety, medication error, etc.), and often with study aims not focused on medication safety preparedness. Existing literature employs diverse methodologies (quantitative and qualitative) and methods (e.g., surveys, interviews) with varying degrees of quality, so substantial gaps remain in the literature worthy of further exploration. Firstly, responsibility for medication management is interprofessional (2, 45), yet previous studies are typically uniprofessional. Secondly, although the broader preparedness literature has started to articulate the psychosocial impacts of unpreparedness [e.g., (46)], and the medication safety literature illustrates the emotional impacts of medication errors for more experienced healthcare professionals (47–51), the medication safety literature is typically silent on the psychosocial impacts of medication errors for new graduates. Finally, previous studies have mostly examined new graduates’ medication safety preparedness at one snapshot in time; rather than their journeys across time [e.g., (7)]. Therefore, we aim to bring additional depth, as well as longitudinal and multiprofessional insights, to explore medication safety preparedness amongst nursing, pharmacy and medicine new graduates, both cross-sectionally (RQ1 and RQ2) and longitudinally (RQ3). Our research questions are:

- RQ1: How do new graduates perceive their medication safety preparedness?
- RQ2: What are the psychosocial impacts of medication errors for new graduates?
- RQ3: How does medication safety preparedness change over and through time?

## Materials and methods

### Study design

This study is part of our longstanding research (46, 52, 53), exploring here preparedness from final-year student to new graduate at a research-intensive Australian University (4, 54–56). This paper focuses exclusively on medication safety preparedness because of its intractable and problematic nature in health professions education, plus we were unable to present this topic in sufficient depth in our previous published work. Our study design is LQR (57–59), underpinned by social constructionism (60). Aligned with temporal theory (55, 59, 61, 62), our findings and recommendations are based on theorizing time in two ways: (a) over time (which conceptualizes time as more objective, linear and fixed); enabling us to compare participants’ experiences at multiple time-points from study outset to conclusion for our whole sample, i.e., before work (around degree completion) and after work (roughly the first 12 weeks of work), and (b) through time (which conceptualizes time as more subjective, fluid and dynamic); enabling us to follow the twists and turns of one participant’s journey, i.e., from week to week through the study phases. We gathered data across three study phases over a 9-month period (July 2019–April 2020): (1) entrance interviews around degree completion (before work); (2) longitudinal audio-diaries (LADs) through approximately the first 12 weeks of work; and (3) exit interviews roughly after 12 weeks of work (63).

### Sampling and recruitment

After receiving ethics approval, employing maximum variation sampling (64), we invited final-year healthcare students through email and in-person invitations, virtual notice boards and social media posts, and snowballing. The sample for this paper comprises 26 final-year students/graduates from nursing, pharmacy, and medicine, with most identifying as female (*n* = 20, 77%) and Oceanian (*n* = 15, 58%). Overall, participant median age was 23.5 years (median of 28 years for nursing, median of 22 years for pharmacy, median of 24 years for medicine).

### Data collection

**FIGURE 1:**
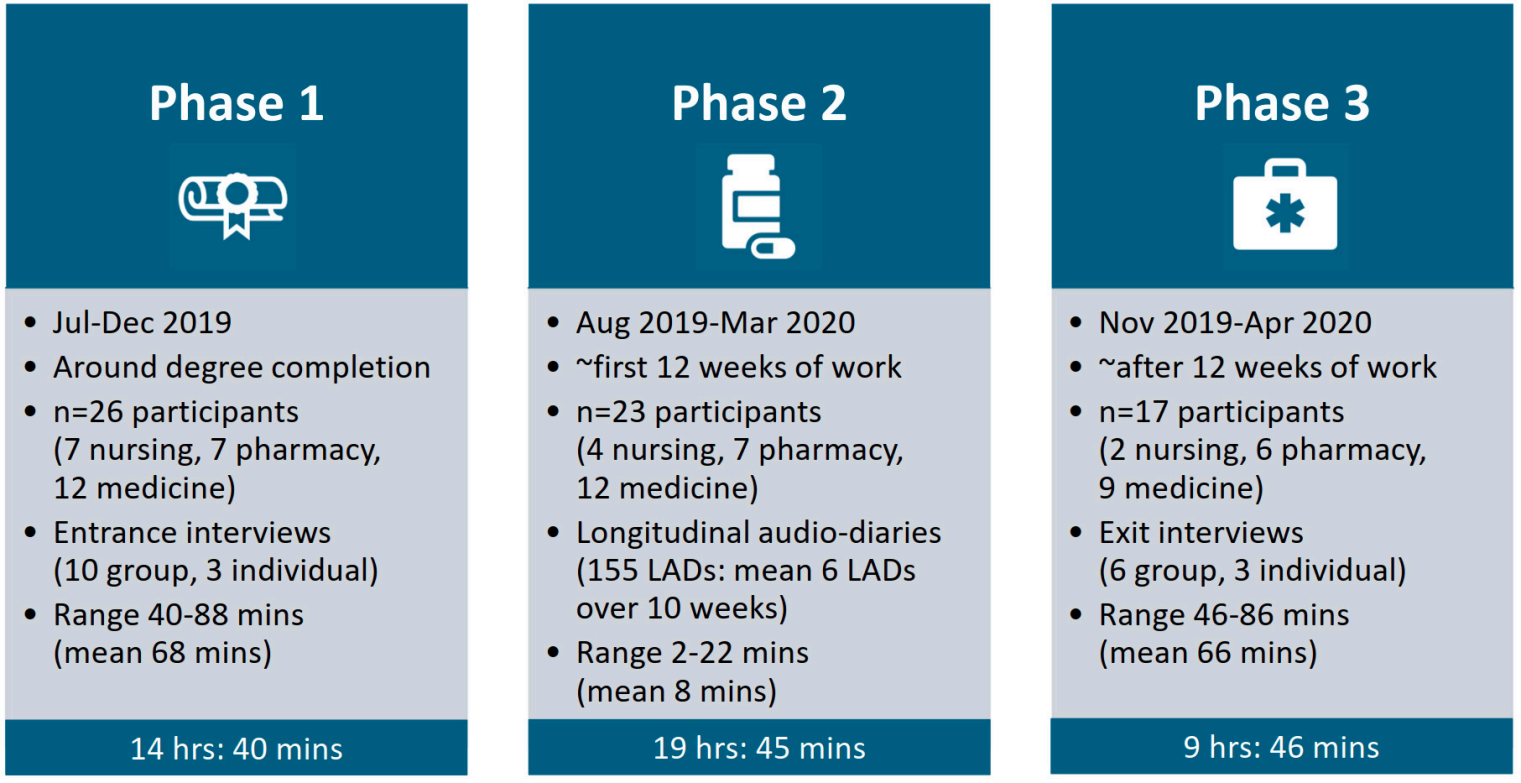
Visual representation of data collection process.

For phase 1, we interviewed 26 participants (*n* = 7 nursing, *n* = 7 pharmacy, *n* = 12 medicine) in ten group entrance interviews (two nursing, three pharmacy, five medicine; 2–3 participants per group), and three individual entrance interviews (nursing). We promoted group interviews but offered individual interviews to maximize participation for comfort or scheduling reasons. Interviews were conducted in-person, led by three researchers (KML, EO and CK–see Section “Acknowledgments”) and audio-recorded. They lasted 68 min on average (range 40–88 min). We flexibly followed a discussion guide developed by our research team, seeking participants’ thoughts on preparedness [e.g., “What does the term “preparedness for practice” (or “work ready”) mean to you?”: (4)], and transitions [e.g., “What does the term “transition” mean to you?”: (56)]. We employed narrative interviewing techniques (65), eliciting memorable stories about times participants felt prepared and unprepared during their final year [e.g., “Can you share with me any memorable experiences from this year where you felt prepared/unprepared for practice,” with prompts: “What happened, in as much detail as you can remember?”; “What did you do?”; “What was the reasoning behind what you did?”; “What were you thinking at the time?”; and “What did you feel at the time?”: (55)]. We concluded the interviews, then invited participants to join phase 2 (see Figure 1 for a summary).

In phase 2, 23 participants (*n* = 4 nursing, *n* = 7 pharmacy, *n* = 12 medicine; 88%) completed longitudinal audio-diaries (LADs) about their preparedness experiences (63). Three researchers (KML, EO and CK) facilitated this phase, each assigned to support the same participants we had interviewed and therefore established rapport with during phase 1. We gave participants an instruction sheet including prompts such as: “Tell us of a time this week when you felt prepared for practice and also a time when you felt less prepared”; “What factors have influenced your preparedness/unpreparedness for practice this week?”; and “How have your preparedness/unpreparedness experiences impacted you this week?” Participants audio-recorded LADs on their own devices, then emailed them to us. We sent weekly reminders, acknowledged LADs received, and asked follow-up questions specific to the content of each LAD. We received 155 LADs in total (1–12 per participant), lasting 8 min on average (range 2–22 min). The number of LADs provided by the different professions varied: 1–12 LADs per nursing participant, 4–12 LADs per pharmacy participant, and 2–11 LADs per medicine participant. We returned LAD transcripts to participants for their own records. LAD participants were invited to join phase 3.

For phase 3, we interviewed 17 participants (*n* = 2 nursing, *n* = 6 pharmacy, *n* = 9 medicine; 74%) in six group exit interviews (two pharmacy, four medicine; 2–4 participants per group), and three individual exit interviews (two nursing, one medicine). These were conducted in-person or via videoconference, led by three researchers (KML, EO and CK), were audio-recorded and followed a similar discussion guide to the entrance interviews, with attention to participants’ longitudinal stories [e.g., “How have your thoughts about preparedness for practice changed (or not) over time, and why?” and “What have been the emotional, psychological and social impacts of your transition experience, and why?”]. They lasted 66 min on average (range 46–86 min). Combined, the three study phases generated over 44 h of qualitative data; which we considered demonstrated sufficient information power (66).

### Data analysis

We conducted team-based framework analysis to enhance the trustworthiness and credibility of our analyses (4, 55, 56, 67). We familiarized ourselves with a selection of deidentified interview and LAD transcripts, independently listening to the audio-recordings while annotating the transcripts, before coming together to develop an initial coding framework. Two researchers (EO and CK) coded transcripts and LAD correspondence in NVivo (Version 12.2.0, QSR International), conferring regularly to develop the coding framework further. Using matrix coding queries, we identified data patterns, comparing by profession and time-point. Time-points explored in our analyses were before work (i.e., data captured in entrance interviews and the participant’s first LAD if it was recorded before starting work) and during work (i.e., data captured in LADs and exit interviews), as well as study week number (e.g., week 1, week 2), with time-points retained and reported in the presentation of our results. We extracted data coded to the medication safety theme (68 pages; 30,205 words) for in-depth analysis. Subsequently, we coded medication management activities, with our codes informed by the five steps of the Pharmacists’ Patient Care Process^1^. Note that some stories were coded to multiple activities, such as assessing medication-related patient information and implementing a medication therapy plan. We also identified and coded medication error stories, where our codes included the error type (commission or omission: see Supplementary Table 1 for medication safety-related codes and code definitions). We also identified emotional talk in these medication error stories, partly because we saw emotional talk as an indicator of preparedness or unpreparedness, but also because we wanted to understand the psychosocial impacts of medication error experiences, as expressed through narrative. We also extracted the preparedness narratives for four longitudinal cases: one nursing (46 pages; 21,350 words), one pharmacy (47 pages; 21,753 words), and two medicine (38 and 44 pages; 17,599 and 20,971 words). Cases were selected based on diary volume and narrative richness, with three already presented in peer-reviewed outputs unrelated to medicines (55, 68). In this paper, we report the nursing case because it has not been presented longitudinally previously, and because of its richness in medication safety narratives. We divided into smaller teams, independently reviewing and annotating extracted data and summarizing our thoughts, before meeting to discuss our interpretations. Finally, we mapped and contextualized our findings by considering the preparedness and medication safety literature, and temporal theory (59, 61). While NVivo enabled us to explore patterns in our data quantitatively, we purposely closeted numbers in our presentation of findings to emphasize our rich qualitative description and qualitative meaning-making (69).

### Team reflexivity

We met regularly throughout the study, drawing upon our diverse expertise, and sharing insights to help achieve our research goals. We completed a team reflexivity activity early in our research (70). Through this, we recognized and appreciated the different views, assumptions and theoretical lenses we contributed, enhancing our interpretation of the study findings. Our authorship team of seven spans five health professions: pharmacy (*n* = 2), psychology (*n* = 2), nursing (*n* = 1), medicine (*n* = 1), and dietetics (*n* = 1). Five of us have clinical experience, six are experienced healthcare educators, and four have medication safety and/or prescribing research interests. Combined, we have extensive qualitative research experience, including with LQR.

## Results

### RQ1. How do new graduates perceive their medication safety preparedness?

Seventeen participants narrated 50 stories about medication safety, including their preparedness (or not) for collecting and assessing medication-related patient information, developing and implementing medication therapy plans, and monitoring and evaluating medication therapy. Some stories contained preparedness and unpreparedness elements. Interestingly, nursing and medicine participants narrated considerably more unprepared stories, while pharmacy participants narrated roughly equal numbers of prepared and unprepared stories. Note that in these results, we refer to our study participants as “participants,” “students” and/or “graduates.” We use the term “participants” for data relevant to students and graduates but we use the term “students” to refer to data from entrance interviews only, and “graduates” for data from LADs and exit interviews only.

#### Feelings of preparedness for medication safety

Prepared stories were typically about assessing medication-related patient information, such as pharmacy graduates completing chart reviews and medication reconciliations, analyzing the appropriateness and effectiveness of prescriptions, and identifying medication errors, drug interactions and adverse reactions, as illustrated by the following female pharmacy graduate (note that we use identifiers to represent our participant data throughout, so in the following quotation, FG16P2P1F, LAD8 means Focus Group 16, Pharmacy Interview 2, Participant 1, Female, with data sourced from Longitudinal audio-diary 8):

*“I’m feeling much better about discharging patients, doing patient medication reconciliation… doing drug interactions, just basic ones, by myself… getting more confident in talking to doctors… I’m getting better at noticing drug interactions…” (FG16P2P1F, LAD8)*

Prepared stories were also about implementing medication therapy plans, including pharmacy graduates processing/dispensing prescriptions and counseling patients, and nursing students safely handling and administering medicines, as described by the following female nursing student:

*“… my preceptor told me that she thought I was ready to take on three patients… One of them… she had a paravertebral infusion… for pain relief… She needed IV antibiotics, because she had pneumonia… it was more about checking her vital signs… then giving her medications on time…” (FG3N2P1F, entrance interview)*

Less often, prepared stories were about developing medication therapy plans, such as pharmacy graduates discussing the appropriate timing/duration of medicines with doctors, and recommending changes to patients’ medications based on pathology or side-effects; and medical graduates prescribing/charting medicines including restricted medicines, as described by the following female medicine graduate:

*“We had a patient who got pretty ill… we had to prescribe him an antibiotic which was restricted… it was very helpful to have gone through prescribing… in the intern orientation… in terms of making sure that I managed to get things done in a short amount of time…” (FG11M2P2F, LAD3)*

Finally, prepared stories concerned collecting medication-related patient information, including pharmacy graduates accessing/reviewing patients’ medical records, pathology and dispensing history, and interviewing patients about their health, medication therapy and history, as explained by the following female pharmacy graduate:

*“… I was doing a medication reconciliation… I was feeling quite confident because I had really read her clinical notes and gone through her medications and her dispensing history, so I knew what questions I wanted to ask. And I felt I conducted the interview really well…” (FG16P2P2F, LAD5)*

#### Feelings of unpreparedness for medication safety

Unprepared stories were predominately about implementing medication therapy plans, such as graduate nurses administering medicines, checking restricted drugs, and making medication errors; pharmacy graduates using unfamiliar dispensing software, making errors when dispensing medicines, and negotiating changes to medication therapy with doctors; and nursing students explaining medicines to patients, prioritizing medication administration, and encountering/administering unfamiliar medicines like blood transfusions and cytotoxic medications, as described by the following male nursing student:

*“It was just a really complex, super complex patient load… I’d never dealt with cytotoxic medications like cancer medications before… learning that whole side of things was another thing that I just got thrown into… It’s just that I wasn’t prepared for that intense or that acute of a setting with such a heavy patient load.” (FG5N4P1M, entrance interview)*

Unprepared stories also concerned developing medication therapy plans, including medical students being unfamiliar with prescribing (including prescribing systems, what to write on drug charts or discharge scripts); pharmacy graduates liaising with doctors about prescriptions, and answering nurses’ questions about medicines; and medical graduates charting medicines to appease patients/nurses, prescribing syringe drivers, and making prescribing errors, as illustrated by the following male medicine graduate:

*“… I was paged by the dietitian to chart a medication… I had to actually chart and rechart the same medication four times, because each time there was a small little mistake… I forgot to add on a different type of medication that went with that medication that I ordered, or had the wrong dose… I was a bit frustrated at myself…” (FG12M3P2M, LAD2)*

Less often, unprepared stories were about assessing medication-related patient information, such as a graduate nurse providing a medicine without considering its clinical implications, and pharmacy graduates not thinking about medications therapeutically, having difficulty annotating medication forms, and using the wrong reference during clinical checks, as explained by the following female pharmacy graduate:

*“… there were other mistakes that I also made… [like clinical checks in] pediatrics, just making sure that it’s okay for their weight, their age, body surface area… So using different references that I hadn’t ever used before… sometimes I got muddled up [and used the wrong reference]… which was obviously a mistake…” (FG17P3P1F, LAD3)*

Finally, unprepared stories were about monitoring and evaluating medication therapy, including medical graduates adjusting medicine doses based on patients’ symptoms or pathology, as described by the following female medicine graduate:

*“… the only thing that really stumped me… I had to… assess and decide the management of a vancomycin dose… the blood levels… were too high… I just called my registrar straight away… we looked up the guidelines together and worked out the dose to change it to…” (FG10M1P2F, LAD6)*

### RQ2. What are the psychosocial impacts of medication errors for new graduates?

As presented in RQ1, we identified numerous medication error stories within participants’ preparedness talk. These stories were coded as prepared or unprepared, or as containing elements of both. Most stories were about participants making errors of commission (doing something wrong) but also included stories about other professionals making errors of omission (not doing something leading to undesirable outcomes). Importantly, these stories contained rich emotional talk, often negative such as anxiety and sadness in the face of medication errors, but sometimes positive. We next summarize the types of errors, and their impacts, as illustrated through participants’ negative and positive emotional talk (see Box 1 for four examples of medication error stories with their associated emotional talk).

BOX 1 **Illustrative quotes for psychosocial impacts of medication errors.**

Quote 1, Unprepared story: “I already make [my] first medication error ever in my first shift. It was a metformin medication… the last nurse forgot to restock the medication… so I need to go to the drug room… I was in a rush, so I took the wrong formula [modified release]… I gave it to my patient without noting anything. So, 2 h later, a pharmacist technician had came and checked every patients’ drawer and she recognized that I have gave the wrong one… they [the pharmacist and medical team] all agree that this is not a big deal, even though it’s still a mistake… so they all happy with the solution that pharmacist will replace the slow release metformin with the immediate release, the correct one, back to the drawer… I talk with all the nurses, and they all said… “mistake happen all the time, so yeah, don’t take it so serious and just learn a lesson from that.” But I was really, really upset about myself, because I feel I was really a bad nurse… I feel really terrible about myself, I nearly cry because I feel so bad…” (FG2N1P2M, LAD1).

Quote 2, Unprepared story: “… I got to spend a bit of time out in the dispensary… I just found the experience just really difficult and really challenging. I was making a lot of mistakes and the pharmacist would have to keep going over and re-dispensing everything… I just was really disappointed in myself and felt really, really hopeless… there were a lot of scripts piling up and customers were having to wait a really long time, it just made things a bit more stressful… I just felt really unprepared and really lost… I didn’t really know what I was doing… when one customer went to the till to pay, he was really confused and really flustered, because the cost of the script was so expensive. And I straightaway knew that I definitely made a bit of a mistake… the pharmacist had to go… and just apologize to the patient and fix up the price.” (FG15P1P2F, LAD4).

Quote 3, Prepared and unprepared story: “… I accidentally injected lignocaine with adrenaline as a digital nerve block, and you’re not supposed to use the adrenaline… I had discussed this with the consultant beforehand, and said specifically that I didn’t want to use adrenaline, but the nurse handed me the lignocaine with adrenaline, and I didn’t check the medication. This was obviously my fault, and I wasn’t prepared for making a mistake. However… it’s been drilled into us that it is normal to make mistakes… and the minor ones, like this one, will be learning experiences for us. And I was prepared in that I knew exactly what to do. I immediately told my consultant, who made sure the patient was okay. I followed open disclosure, so I told the patient what had happened and that I was very sorry… I had to fill out a Riskman [incident report]… And then had a chat to the consultant the following day and he just said, “Look, basically what you take out of this is that you check your medications”… all in all… for a very shocking experience, it was actually a good one. It’s taught me a very valuable lesson…” (FG10M1P2F, LAD4).

Quote 4, Prepared story: “… when I did do stuff by myself and accomplish things, I guess I was proud of myself. So, I was checking a chart for one of the patients that my supervising pharmacist had given to me… And I was checking her chart… in her notes previously [she] had been taking a dose of Mirtazapine… at night to help her sleep. It was a dose of I think about 30, maybe even 45 mg, which is like a usual sort of dose at night when required. And when checking her chart, I saw that she had been charted for 150 mg at night, and I was like, “Whoa, that’s quite a big dose increase.” It was actually a mistake and had to get amended by the doctor to change that dose, because it would have not been good for the patient. So, I was proud of myself for picking that one up.” (FG16P2P2F, LAD11).

#### Types of errors

Participants recounted stories about making medication errors themselves. For nursing graduates, errors included giving the wrong medication (Box 1, quote 1) or the wrong dose, giving wrong information about a medication, and failing to withhold a charted medication based on patient observations. For pharmacy graduates, errors related to using the wrong reference, mistakes in dispensing processes, and incorrectly pricing medications (Box 1, quote 2). For medicine graduates, errors included making mistakes on drug charts, failing to chart required medications, and injecting a patient with the wrong medication (Box 1, quote 3). Participants also recounted stories about other professionals making medication errors. These were predominately interprofessional; pharmacy graduates identifying errors in doctors’ prescribing, such as doctors charting the wrong drug or dose, charting suboptimal duration or timing, failing to chart a required medication, and failing to dose reduce or cease a medication (Box 1, quote 4). Concerning error resolution, graduates spoke about reporting errors to seniors, and seeking their reassurance/guidance (Box 1, quote 2). Interprofessional discussions were also identified, such as nursing graduates consulting pharmacists and medical teams (Box 1, quote 1), and pharmacy graduates making recommendations to medical prescribers (Box 1, quote 4). Additionally, nursing and medicine graduates described monitoring patients, following open disclosure, and reporting through risk management systems (Box 1, quote 3).

#### Negative emotional talk

Some graduates minimized medication errors, talking about them as: “small,” “stupid” or “silly” mistakes, or by explicitly saying the error: “wasn’t a big thing” or “minor” (Box 1, quote 3). Others catastrophized errors, reporting wide-ranging emotional responses, including anxiety (e.g., panic/stress, fear/worry), anger (e.g., frustration/annoyance), and sadness (e.g., hopelessness). Nurses’ anxiety talk seemed dominant in the data, with them expressing feeling: “really stressed,” “really worried,” “panicking” and going “limp” when they erred (see Table 1, stories 6 and 7 later). They also expressed their sadness (e.g., “really, really upset,” being close to tears) and anger (e.g., “annoyed” at themselves, cursing themselves for “really careless” mistakes, labeling themselves derogatorily, e.g., “bad nurse”: Box 1, quote 1). They recognized their anxiety about making medication errors, with one participant admitting to dreaming about their whole ward dying (see Table 1, story 7 later). Pharmacy graduates also expressed feeling anxious (e.g., “stressful”) and sadness (e.g., “really disappointed” in themselves, “really, really hopeless” about their dispensing errors: Box 1, quote 2). They also described feeling: “really lost” and not knowing what they were doing. While medicine graduates also employed some sadness (e.g., “very sorry”) and anger talk (e.g., “a bit frustrated at myself”), they appeared to report less anxiety talk, instead identifying feeling: “pretty calm” and “[not] too panicked” when they erred.

**TABLE 1:** Xanthia’s medication safety stories through time.

#	Time^a^	Story focus	Illustrative quotation from narrative
1	Before (week 0)	Unprepared story about prioritization of care involving medications	“… I’m not prepared to be doing this [prioritization of blood transfusion then IV antibiotics] on my own… that was pretty scary [laughs]… it’s just crazy and I’m not… prepared to be able to do that prioritization… Frustrated I suppose and very scared about what’s coming [laughs]… How on earth am I going to do it on my own?” (FG4N3P1F, entrance interview).
2	Before (week 0)	Unprepared story about medication knowledge	“… medication is everyone’s scary part… my pharmacology knowledge is not that good… If I was good at it, then I would actually not have to stress… I’m scared. If someone [a patient] asks me a question… will I be able to answer that?… you can’t have that [leniency to not know as a student] when you’re a grad registered nurse [laughs].” (FG4N3P1F, entrance interview).
3	During (week 1)	Unprepared story about medication administration	“Being accountable and responsible for people’s health and lives is a big transition… if I make a mistake while I’m checking [a restricted drug with another nurse], then I’m also liable… so that’s very scary… giving out medications on my own was quite nerve-wracking. I was taking a long time because I was just checking the medications three times… I was petrified that I would make a mistake.” (FG4N3P1F, LAD1).
4	During (week 2)	Prepared and unprepared story about medication administration	“… I’m getting a little bit more confident in administering medications… the normal medications… I’m getting a bit more confident with asking for schedule 11 [controlled] drugs… However, I still feel very scared to do IV medications… which is really silly… I haven’t had enough experience to be competent and confident… I still get freaked out when the pump makes a noise… I feel very incompetent…” (FG4N3P1F, LAD2).
5	During (week 7)	Prepared story about medication administration diligence	“[My appraisal meeting] went really well. Sat down with my nurse unit manager… she said, “I’ve noticed that you are super cautious of your medications, to an extent where you are obviously running late… But that’s okay. I would rather you be cautious than make an error because you are trying to rush”… everything has been marked as meeting expectations [for a first rotation graduate]… so that’s really good…” (FG4N3P1F, LAD7).
6	During (week 8)	Unprepared story including medication error of commission	“… I was doing my 8am medications and I gave a patient all his medications… After 4 h… his heart rate was 52… I asked my buddy to come and double check… she asked me… “Has he had any heart medications?”… it just hit me… I was like, “Shit. I think I gave it [Metoprolol] to him when his heart rate was low already”… I started panicking… that was such a stupid mistake… I was just cursing myself… I went back to my buddy nurse… and she’s like, “You’re stressing too much. It’s going to be fine…” So then after maybe an hour or so… I went and checked his heart rate again, and he was up to normal, and then I felt a bit better… being tired affects your… attentiveness and makes it easy to make mistakes… I just felt… I’ve done so many 8 o’clock medications, now I’m just dishing up medications without thinking?… I know it wasn’t a major disaster, but… I need to be… on the ball with these medications… So that was a bit of an eye-opener, a good learning experience… I was really stressed…” (FG4N3P1F, LAD8).
7	During (week 11)	Unprepared story including medication error of commission	“… [the patient] was still very unwell. She had a PICC line, she had IV antibiotics… I was just running around like a blue-arsed fly, getting things done… I hadn’t really worked with PICC lines before… while I was doing the medication, she [the patient] was talking to me… we have to do the five rights of medication… I was looking at the dose, and it said 2 mg… I didn’t even realize that I had actually popped two pills… And… she [the patient] did question me… I didn’t pay attention to her when she said she only had one tablet… Then as soon as she took it… then it hit me, and I checked it and… I just went limp… I was really worried… I went to my nurse-in-charge… She was really kind… Then… I had to tell my patient… she wasn’t super upset… But… I was pretty upset with myself… I was annoyed at myself that I wasn’t paying attention, that… I didn’t listen to my patient… And then… I went home and on Friday night I dreamt that my whole ward died… I guess I was very anxious and worried about the medication error… I guess my lesson learned is to be a bit more careful, and… definitely listen to my patient… I just ignored her, which is really terrible…” (FG4N3P1F, LAD11).
8	During (week 16)	Prepared and unprepared story about priming IV lines	“… I remember when I first started to prime the IV line, I would probably take forever… I was not good with my hands… I was worried about what I’m doing… But now… I’ve done enough to know, I’ve got my own little technique… which I’ve picked up from other people, on how to prime it properly… and when to prime it… So, I guess the more exposure I’ve been getting and the more actually I’ve been doing it myself, definitely helped being comfortable…” (FG4N3P1F, exit interview).
9	During (week 16)	Unprepared story about medication knowledge	“… I was definitely not prepared for medications… my pharmacology knowledge wasn’t great then [before work], and it’s not great now [approximately 16 weeks into work]… but… now because I’m independent, I feel more responsible… I have easy access to… look up what the medication is. So… I feel a little bit more comfortable… And… the more I’m exposed to the same medications, then I guess my knowledge about them gets a bit better… I still would like to be a bit more knowledgeable… I want to be able to explain things to a student… Every time a student asks me, I’m like, “Don’t come with me. You’ll drown… I’m drowning and I’ll drown you with me” [laughs]… I think I’ll just have to study [to improve]… And… the more common the medications… the more times I administer it… it starts sticking in my head…” (FG4N3P1F, exit interview).

*^a^*Before = before work; During = during work.

#### Positive emotional talk

Graduates also employed positive emotional talk when recounting medication errors. For example, one nursing graduate described their experience as: an “eye-opener” and a “good learning experience” (see Table 1, story 6 later). Another pharmacy graduate narrated having: “learnt a lot by doing it.” Interestingly, pharmacy graduates repeatedly employed positive emotional talk in their medication error stories. For example, talking about feeling a sense of accomplishment and being proud of themselves when they identified mistakes by other professionals on patients’ prescriptions or drug charts (Box 1, quote 4), or when they reached their 100 dispense checklist without making a mistake. One pharmacy graduate also described a patient being thankful and a doctor being grateful for their intervention. Finally, one medicine graduate described their experience as simultaneously “very shocking” but “good,” teaching them a “very valuable lesson” (Box 1, quote 3). They also expressed how it was normal and expected of them to make mistakes, viewing medication errors as learning opportunities.

### RQ3. How does medication safety preparedness change over and through time?

Across the cohort, we collected more medication safety stories from graduates (i.e., during work) than from students (i.e., before work). As a cohort, while the stories were mostly unprepared before and during work, the proportion of prepared stories increased over time. However, prioritizing understandings of time as fluid and dynamic, we were able to identify more nuanced preparedness patterns through time by exploring longitudinal data at the individual level. Here, we identified four participants narrating medication safety stories before and during work, with either stability or different patterns of change evident in their preparedness (note that these participants are not necessarily the same as the four longitudinal cases described in the methods). For example, one nursing graduate showed persistent unpreparedness across time, another nursing graduate showed a negative shift from preparedness before work to unpreparedness during work, and one medical graduate showed a positive shift from unpreparedness before work to preparedness during work (despite expressing unpreparedness again at the study conclusion after roughly 12 weeks of work). The fourth participant (our longitudinal case)–a nurse called Xanthia (pseudonym)–articulated a positive shift toward preparedness over time:

*“[I’m] definitely more prepared than when I started. Four months ago, I was not prepared. Now I feel more prepared…” (FG4N3P1F, exit interview)*

However, longitudinal analysis of her medication safety stories indicates more complex preparedness patterns through time. So, we present the analysis of her in-depth longitudinal case next. She participated in an entrance interview around degree completion, 12 LADs over 14 weeks, and an exit interview at week 16, generating 3 h and 17 min of data (weeks 0–16). Xanthia recounted student stories from her final-year community and hospital placements, and during work as a graduate nurse in a metropolitan health service. As a new graduate, Xanthia rotated through a general medical ward, followed by a specialist medical ward. In relation to medication safety, Xanthia narrated nine stories across time (see Table 1 for a longitudinal summary).

Before starting work, Xanthia talked about her unpreparedness for medication management, describing her fear of autonomy (Table 1, story 1), and lack of medication knowledge (Table 1, story 2). In her first week of work, Xanthia reported grappling with increased accountability and responsibility, nerves when administering medications, and taking her time to avoid making medication errors (Table 1, story 3). Indicating some improvement in week two, Xanthia talked about her increasing confidence with some aspects of medication management (e.g., medication administration, restricted drugs), but not others due to her perceived lack of experience (e.g., IV medications: Table 1, story 4). Despite expressing positive emotional talk by week seven (e.g., “really good,” “really well”) in relation to supervisor feedback about her cautious/diligent approach to medication administration (Table 1, story 5), Xanthia later narrates two stories involving medication errors in weeks eight and eleven (Table 1, stories 6 and 7). In both stories, she talked about her tiredness and inattention, acknowledging error contributory factors, such as busyness and distraction. These errors, as well as her medication experiences more broadly, were stressful and upsetting experiences for Xanthia, as evidenced by her anxiety (e.g., panic, worry, scary, nerve-wracking, petrified, anxious) and anger talk (e.g., annoyed, cursing herself, frustrated), her repeated laughter (probably for coping), as well as her metaphoric talk emphasizing life-threatening danger (e.g., “went limp,” “it just hit me,” “drowning”). She described her learnings from these experiences, including the need to demonstrate more care and attention regarding medications. At the study conclusion, in week sixteen, Xanthia reflected on her improved preparedness, comfort and competence with priming IV lines, following more experience and practice (Table 1, story 8). She also seemed more accepting of her independence, indicating a positive shift in her views on autonomy (Table 1, story 9). However, despite increased exposure and experience with administering medications, and accessible resources, Xanthia reported persistent feelings of unpreparedness regarding her medication knowledge (Table 1, story 9). When asked in her exit interview about her development over time, Xanthia commented on her improved familiarity with medicines:

*“… I’m becoming more familiar with medications… I don’t have to look up a medication every single time… when I see it, I know what it is for…” (FG4N3P1F, exit interview)*

In her exit interview, she also talked about becoming more comfortable with practicing autonomously:

*“I think what I’ve realized in the 4 months is the good thing about looking after my patients on my own… yes, I have that responsibility of four people, which is scary, but at the same time, I don’t have someone behind me, watching me all the time. And that’s a comfort… when I was a student… I was always with a buddy nurse… I had to ask them for everything… “I’m going to do this, what do you think?” … or I needed her to be another nurse… if I had to give medications… But I quite find the autonomy helpful… it forces me to do things properly…” (FG4N3P1F, exit interview)*

## Discussion

Our study explored medication safety preparedness amongst an Australian sample of nursing, pharmacy and medicine graduates employing a novel LQR approach to answer two cross-sectional and one longitudinal research questions. We next summarize our key findings by RQ and discuss them by comparing them with existing research, especially highlighting how our findings extend and deepen current literature. We hope to show how our findings bring depth, as well as longitudinal and multiprofessional insights, to the existing broad, disparate and fragmented literature relating to medication safety preparedness.

### Summary of key findings and comparisons with literature

Regarding RQ1, participants’ medication safety stories demonstrated mostly unpreparedness, consistent with earlier uniprofessional research [e.g., (7, 9, 18)]. Unprepared stories in our study were mostly about developing and implementing plans for medication therapies, aligned with some uniprofessional research [e.g., (11)]. These findings are perhaps unsurprising given that our data were collected from participants around the time of degree completion and into their first 12 weeks of work [e.g., (71)]. However, that we still find such reported unpreparedness suggests that participants’ undergraduate and early workplace-based training remains suboptimal, especially around the development and implementation of medication therapy plans [e.g., (23, 30, 42)]. However, participants also narrated preparedness, as has been found in earlier uniprofessional research [e.g., (28, 30)]. Furthermore, prepared stories were largely about assessing patients’ medicine-related information and implementing medication therapy plans, also consistent with some uniprofessional research [e.g., (28)]. These preparedness findings suggest that some elements of participants’ undergraduate and early workplace-based training such as information gathering connotes preparedness benefits [e.g., (28)]. Interestingly, given our unique multiprofessional study, pharmacy participants seemed to narrate more medication safety preparedness stories than nursing and medicine participants, thereby extending and deepening existing uniprofessional literature. This finding could reflect pharmacy graduates’ satisfaction with their preparation for assessing patients’ medicine-related information and dispensing, or it could simply reflect the broader focus of our study on general preparedness (with therefore more of pharmacy participants’ preparedness narratives being about medication safety than those of nursing and medicine participants). Also extending and deepening this uniprofessional literature are our narrative-orientated findings that medication safety stories sometimes included elements of preparedness and unpreparedness, thereby illustrating the complexity of new graduate medication safety preparedness experiences–in just one situation, for example, a new graduate might feel simultaneously prepared for one aspect (e.g., prescribing) but unprepared for another (e.g., making a prescribing mistake). So, our narrative approach serves to illustrate the contextual nuances of preparedness situations for students and new graduates, demonstrating that events are not always straightforward preparedness or unpreparedness examples (71).

Relating to RQ2, participants recounted numerous medication error stories, consistent with previous research [e.g., (8)]. These narratives were rich in mostly negative emotional talk; talk which could be seen as an indicator of unpreparedness, as well as illustrating the negative psychosocial impacts on new graduates. While such negative emotion has been found in junior doctors’ general unpreparedness stories [e.g., (46)], our study extends this literature by illustrating the interplay between medication safety preparendess and emotion. Anxiety, sadness and anger talk were commonplace in medication error stories, as has been found in research with more experienced professionals [e.g., (47–51)]. However, given our unique multiprofessional study, we also noticed that medical graduates seemed to employ less anxiety talk in their narratives than nursing and pharmacy graduates. While this distinction may reflect differences in the emotional preferences of our individual participants, it could also reflect variations in training across the professions. For example, one of our medical participants (FG10M1P2F, LAD4) implied that their training normalized mistakes and viewed them positively as learning opportunities (“… [it was] drilled into us that it is normal to make mistakes… and the minor ones… will be learning experiences). Indeed, error stories sometimes included positive emotional talk in relation to learning through mistakes, as well as pharmacy graduates identifying and rectifying others’ mistakes. These findings are largely absent from, and therefore serve to extend previous literature on medication errors with experienced professionals. We think that experienced professionals are less likely to see the positives in mistakes, possibly because they no longer see themselves as learners, and consequently have higher expectations on themselves to practice medication safety, and prevent medication errors.

Finally, regarding RQ3, participants mostly narrated unprepared stories before and during work, but the proportion of prepared stories increased at the cohort level across time. This is consistent with a recent longitudinal cohort study (7), which found that nursing students were mostly unable to verify all five rights at graduation, although the numbers verifying all five rights increased from 0% in semester 1 compared to 20% in semester 4 (i.e., graduation). At the individual level, however, we found a more nuanced/complex patterning in participants’ narratives including evidence of stability, positive change, or negative change. This finding is also consistent with another longitudinal narrative study with junior doctors (71), which similarly illustrates the complexities and nuances of preparedness shifting through time. However, this study did not tease out medication safety preparedness specifically, so our study serves to extend and deepen existing literature. Furthermore, our longitudinal data (including our in-depth longitudinal case analysis) illustrates the highly individualistic, non-linear, iterative and ongoing medication safety preparedness journeys during the early new graduate phase, with its metaphorical twists and turns, and a sense that new graduates have considerable medication safety preparedness journeys ahead of them.

### Methodological strengths and challenges

Regarding strengths, firstly, our study demonstrates internal coherence (72), with alignment between our study’s philosophy (interpretivism, constructionism), methodology (e.g., LQR, narrative), and methods (e.g., maximum variation sampling, interviews, LADs, cross-sectional and longitudinal analyses). Our study conduct is also consistent with other LQR in health professions education research (59, 73), and our reporting aligned with the Standards for Reporting Qualitative Research (74). Secondly, our sampling included multiple professions, addressing a gap in the uniprofessional literature (21, 36), as well as close-noticing of differences between the professions in terms of medication safety experiences and their narrated emotions. Furthermore, our sample had adequate information power, with 26 participants contributing over 44 h of data (66). Finally, our novel LQR approach collecting narratives through interviews and LADs, alongside our rigorous and reflexive team-based analysis enabled us to better understand the emotional impacts of medication errors on new graduates, as well as appreciating individual medication safety journeys over and through time.

Our study is not without its limitations however, and these should be considered when interpreting our findings and providing recommendations. First, our interview questions and LAD prompts did not specify medication safety preparedness *per se*. While this gives a certain authenticity to our (unsolicited) data, we did not probe these medication safety narratives further, potentially leaving gaps in our analytic understanding of new graduates’ experiences such as participants’ coping mechanisms in the face of medication errors. Second, our dataset is modest, partly because our participants provided diverse amounts of data (e.g., number and length of LADs), but also because we did not ask specifically for medication safety preparedness stories. This meant we received voluminous medication safety data from some participants preoccupied with these experiences, but very little from others. Therefore, while it was possible to provide some longitudinal cohort findings in this paper, as well as one in-depth longitudinal nursing case, it was not possible to present additional longitudinal cases for medicine and pharmacy. Furthermore, given this modest dataset, caution is required with our close-noticing and reporting of differences in narratives provided by participants from different professions. Third, we experienced various pragmatic issues potentially affecting the transferability–applicability, resonance and theoretical engagement–of our study findings (75), where: (a) we observed attrition amongst our nursing participants across time (*n* = 7 at phase 1, *n* = 4 at phase 2, and *n* = 2 in phase 3), meaning that our cross-sectional and longitudinal findings may resonate suboptimally with other new graduate nurses; (b) our study was conducted with Australian graduates, meaning that our findings may not be transferable to graduates from other countries with different undergraduate programs or postgraduate workplace structures or cultures; (c) our study duration was relatively modest (the first 12 weeks of work), meaning that our data are only applicable to the early new graduate transition; and (d) our study did not engage with any specific micro/macro preparedness theories to frame or explain our findings, although we engaged with grand temporal theory throughout our broader LQR study (discussed earlier in the section “Materials and methods”) (55). Finally, although our study was multiprofessional, we interviewed participants in uniprofessional groups (for reasons of participant comfort and ease of scheduling), plus we did not ask participants specific interprofessional questions. Although we collected some interprofessional narratives from participants, we missed opportunities to more fully explore interprofessional medication safety preparedness.

### Research implications

Based on these methodological challenges, we recommend further LQR focusing specifically on medication safety preparedness in diverse country settings, enabling a deeper appreciation of new graduates’ experiences and transitions. LQR should include multiple professions like our study (particularly nursing, pharmacy and medicine graduates) with larger numbers of participants, but unlike our study could include interprofessional group interviews (rather than uniprofessional), and ask specifically for interprofessional medication safety stories, to gain a better understanding of interprofessional education opportunities (45). Perhaps most importantly, further LQR should include a longer study duration to elucidate new graduates’ medication safety preparedness journeys beyond the early phase covered in this study. Funding permitting, we would recommend a minimum data collection duration of 18 months, for example, starting in final year and extending to (or just beyond) the first year of work.

### Educational implications

Our study indicates that new nursing, pharmacy and medicine graduates would benefit from additional formal and informal learning opportunities at undergraduate and postgraduate levels, to build their preparedness for developing and implementing medication therapy plans, as well as their preparedness for the prevention and management of medication errors, possibly employing interprofessional simulation and workplace-based learning activities, as suggested by others (15, 21, 36). However, our findings tentatively suggest additional content ought to be covered as part of any formal and informal medication safety education at undergraduate and postgraduate levels. Firstly, medication safety education interventions should cover the emotional aspects of medication management activities, helping new graduates prepare for, and manage, the positive and negative emotions they will inevitably feel, especially in the face of medication errors. Secondly, medication safety education interventions should help final year students and new graduates better understand the individualistic, non-linear, iterative, and ongoing developmental aspects of medication safety preparedness, to ensure that they appreciate the inescapable messiness of their own preparedness journeys. New graduates need to appreciate that they will sometimes feel simultaneously prepared and unprepared for medication management, and that their medication preparedness journeys may seem to head in the right direction sometimes, but at other times in the wrong direction, and that that is acceptable. We recommend that interested educators weave this content into existing or extended medication safety curricula as they see fit. However, consistent with our research methods, we advocate for narrative and longitudinal educational approaches, such as reflexive longitudinal audio-diaries to help learners make sense of their medication safety journeys, including the emotional twists and turns along the way. We think that any educational interventions could include papers like ours as recommended reading to new graduates so they can understand the uniqueness of their own journeys but also realize that they are not alone in their struggles–the path is well-worn. With these additional scaffolded learning opportunities, new graduates will hopefully feel better able to cope with their medication management activities including errors, for the betterment of themselves, and ultimately patients.

## Data Availability

The datasets presented in this article are not readily available because the raw data supporting this article’s conclusions cannot be made available by the authors, because they do not have ethical approval to share these data. Further inquiries can be directed to the corresponding author, charlotte.rees@swansea.ac.uk.

## Footnotes

1
https://jcpp.net/patient-care-process/
