# Supplementary material for: New graduate medication safety preparedness: an Australian cross-sectional and longitudinal qualitative research study
Source: Front Med (Lausanne). 2026 Feb 13;13:1704787. doi: 10.3389/fmed.2026.1704787 (PMC12945809; doi:10.3389/fmed.2026.1704787)
Supplement: Supplementary file 1 [file Table_1.docx]

**Online Supplementary Table 1: Codes and code definitions for medication safety data^a^**

| **Code** | **Code definition** |
| --- | --- |
| Collecting medication-related patient information^b^ | Participants collecting patient information, including medical history, medication history or patient clinical status, from sources such as the patient/caregiver, the medical record or a healthcare professional |
| Assessing medication-related patient information^b^ | Participants assessing patient information and analysing therapy effects on health to optimise patient care, including chart review and medication reconciliation |
| Developing medication therapy plan^b^ | Participants developing a medication therapy plan in collaboration with the patient/caregiver or a healthcare professional, including prescribing or charting medicines |
| Implementing medication therapy plan^b^ | Participants implementing a medication therapy plan in collaboration with the patient/caregiver or a healthcare professional, including dispensing or administering medicines, medicine-related education or training |
| Monitoring and evaluating medication therapy^b^ | Participants monitoring and evaluating a medication therapy plan, including modifying the plan in collaboration with the patient/caregiver or a healthcare professional |
| Error of commission – wrong drug/dose | Giving the wrong drug or dose |
| Error of commission – wrong information | Giving the wrong information about a drug |
| Error of commission – dispensing | Making a dispensing error, such as wrong pricing |
| Error of commission – drug chart | Making an error on the drug chart, such as charting the wrong drug or dose |
| Error of commission – overmedicating | Overmedicating, such as charting a drug to appease patient |
| Error of commission – wrong reference | Using the wrong reference, such as during clinical checks |
| Error of omission – failure to prescribe | Failing to prescribe the optimal dose, duration or timing |
| Error of omission – failure to chart | Failing to chart a required drug |
| Error of omission – failure to administer | Failing to administer a drug correctly, such as poor inhaler technique |
| Error of omission – failure to withhold/adjust | Failing to withhold or adjust a drug, such as dose reduce or cease a drug |

^a^Medication safety theme defined as: “Participants describe feeling prepared to prescribe, administer and dispense medications/IVs, as well as monitor/evaluate medication management.”

^b^Adapted from the Pharmacists’ Patient Care Process (<https://jcpp.net/patient-care-process/>)
